# Altered static and dynamic functional network connectivity in post-traumatic headache

**DOI:** 10.1186/s10194-021-01348-x

**Published:** 2021-11-13

**Authors:** Fengfang Li, Liyan Lu, Song’an Shang, Huiyou Chen, Peng Wang, Vijaya Prakash Muthaiah, Xindao Yin, Yu-Chen Chen

**Affiliations:** 1 Department of Radiology, Nanjing First Hospital, Nanjing Medical University, No.68, Changle Road, 210006 Nanjing, China; 2 Department of Rehabilitation Science, School of Public Health and Health Professions, University at Buffalo, Buffalo, USA

**Keywords:** Post-traumatic headache, Mild traumatic brain injury, Functional magnetic resonance imaging, Functional network connectivity

## Abstract

**Background:**

Post-traumatic headache (PTH) is a very common symptom following mild traumatic brain injury (mTBI), yet much remains unknown about the underlying pathophysiological mechanisms of PTH. Neuroimaging studies suggest that aberrant functional network connectivity (FNC) may be an important factor in pain disorders. The present study aimed to investigate the functional characteristics of static FNC (sFNC) and dynamic FNC (dFNC) in mTBI patients with PTH.

**Methods:**

With Institutional Review Board (IRB) approval, we prospectively recruited 50 mTBI patients with PTH, who were diagnosed with ICHD-3 beta diagnostic criteria and 39 mTBI without PTH who were well matched for age, gender and education. Resting-state functional magnetic resonance imaging (fMRI) scanning (3.0 T, Philips Medical Systems, Netherlands), Montreal Cognitive Assessment (MoCA) and headache symptom measurement (headache frequency and headache intensity) were performed. The resting-state fMRI sequence took 8 min and 10 s. Independent component analysis and sliding window method were applied to examine the FNC on the basis of nine resting-state networks, namely, default mode network (DMN), sensorimotor network (SMN), executive control network (ECN), auditory network (AuN), attention network (AN), salience network (SN), visual network (VN), and cerebellum network (CN). The differences in sFNC and dFNC were determined and correlated with clinical variables using Pearson rank correlation.

**Results:**

For sFNC, compared with mTBI patients without PTH, mTB with PTH group showed four altered interactions, including decreased interactions in SN-SMN and VN-DMN pairs, increased sFNC in SN-ECN and SMN-DMN pairs. For dFNC, significant group differences were found in State 2, including increased connectivity alteration in the DMN with CN, DMN with SMN, and AuN with CN. Significant reduced connectivity changes in the DMN with VN was found in State 4. Furthermore, the number of transitions (r=0.394, *p*=0.005) between states was positively associated with headache frequency. Additionally, dwell time (r=-0.320, *p*=0.025) in State 1 was negatively correlated with MoCA score.

**Conclusions:**

MTBI patients with PTH are characterized with altered sFNC and dFNC, which could provide new perspective to understand the neuropathological mechanism underlying the PTH to determine more appropriate management, and may be a useful imaging biomarker for identifying and predicting mTBI with PTH.

## Introduction

Post-traumatic headache (PTH) is a highly disabling secondary headache disorder and one of the most common sequelae of mild traumatic brain injury (mTBI)[1]. The prevalence of PTH is reported as high as 79 % at 3 months and 65 % at 12 months after mTBI[2]. PTH can be attributed to mTBI or moderate or severe traumatic brain injury, and PTH is often accompanied by mood, cognitive autonomic and sleep symptoms[3]. However, the underlying neuropathological mechanism of PTH after mTBI still remains unknown. Therefore, further work is needed to investigate the mechanisms of acute PTH and PTH persistence following mTBI, and to determine the specificity of the imaging findings for PTH.

Resting-state functional MRI was used to quantify brain functional connectivity (FC) and functional organization[4]. Previous studies have identified aberrant FC within networks including the default mode network(DMN), executive control network(ECN), salience network(SN) and visual networks(VN) in mTBI [5–7]. Furthermore, significant FC differences between migraine and mTBI with persistent PTH were found [8–10], and the altered functional network connectivity (FNC), such as DMN-attention network(AN) and VN-AN, were identified in mTBI patients[11]. Overall, these results suggest that network alterations reflect clinically relevant phenomena in mTBI and mTBI with PTH. However, most of the previous studies did not take into account important dynamic aspects that change over time.

Static FNC (sFNC) analysis ignores the fact that individual subjects may have slightly different psychological activities in different situations at different times[12]. In addition, emerging evidence indicates that the brain is a complex system with dynamic properties and time-dependent [13]. Recently, studies have begun to take advantage of the powerful information contained in the temporal characteristics of the spontaneous FNC of the blood-oxygenation-level dependent (BOLD) signal [14]. Alterations in dynamic FNC(dFNC) are associated with specific psychiatric conditions, cognitive states, and neurological diseases[15–17]. In addition, studies of mTBI have demonstrated potential biomarker utility and the clinical relevance of dFNC[18, 19, 11]. However, alterations in dFNC are still largely unknown in mTBI patients with PTH.

The present study aimed to investigate the difference of sFNC and dFNC between the mTBI with and without PTH, using resting-state fMRI and sliding-window analysis. We hypothesized that the sFNC, dFNC, and the temporal properties of dynamic FC states would characterize the underlying nature of mTBI patients with PTH.

## Materials and methods

### Participants

This was a prospective study design and the Institutional Review Board (IRB) approval was obtained. A total of 89 mTBI patients participated in this study. MTBI was defined according to the American Congress of Rehabilitation Medicine[20]. Inclusion criteria were as follows: (a) patients aged 20 or older; (b) initial Glasgow Coma Score (GCS) of 13–15; (c) loss of consciousness<30 min; and (d) post-traumatic amnesia<24 h. Exclusion criteria were as follows: (a) a history of previous head injury; (b) history of pre-existing psychiatric or neurological disease; (c) history of illicit drug or alcohol abuse; (d) dental appliances that might distort the functional MR images; (e) history of migraine or any other headache prior to injury. and (f) MRI contraindications. Given the emergency care setting, it was not feasible to perform a full battery of cognitive assessments. Therefore, the clinical neurocognitive state of all participants of psychosis was quantified with the Montreal Cognitive Assessment (MoCA)[21], which is a sensitive cognitive screening test following mTBI and only requires limited training to administer. All subjects underwent the same MRI scan and cognitive function assessment within 0-7 days after trauma. In addition, none of the patients were receiving medication for headache. The present study was approved by the local ethics of Nanjing Medical University. Written informed consent were obtained from all participants before undergoing MRI.

### Headache symptom measurement

After 12 months, all headache diagnoses were confirmed by two experienced headache specialists using International Classification of Headache Disorders 3rd edition, beta version (ICHD-3 beta) diagnostic criteria [22]. MTBI patients with PTH provided detailed information about the headache, including the main location, headache intensity, and headache frequency (day/month). All participants completed the visual analogue scale (VAS)[23], a numerical scale from 0 to 10, with 0 representing no pain and 10 representing the most severe pain imaginable, to report the intensity of their headache.

### MRI data acquisition

Images were acquired on 3.0 T Philips Ingenia scanner (Philips Medical Systems, Netherlands) using an 8-channel head coil. The subjects were asked to lie quietly with their eyes closed, not to fall asleep, not to think about anything in particular, and to avoid any head movement during the scanning. Structural 3D T1-weighted images were acquired with the three- dimensional turbo fast-echo (3D-TFE) T1WI sequence with the following specifications: repetition time (TR)/ echo time (TE) = 8.1/3.7 ms; slices = 170; gap = 0 mm; thickness = 1 mm; FA = 8°; FOV = 256 mm × 256 mm; and acquisition matrix = 256 × 256. Functional images were acquired using a gradient echo-planar imaging sequence with the following parameters: TR / TE = 2000/30 ms; slices = 36; gap = 0 mm; thickness = 4 mm; field of view (FOV) = 240 mm × 240 mm; acquisition matrix = 64 × 64; and flip angle (FA) = 90°. A single resting-state fMRI run lasted for 8 min and 10 s. The susceptibility weighted imaging (SWI) used a 3D gradient echo (GRE) sequence with the following parameters: TR/ TE = 22 /34mm; FA = 20; slice thickness = 1 mm; matrix = 276 × 319; and FOV = 220 mm ×220 mm. The specifications for fluid-attenuated inversion recovery (FLAIR) were as follows: TR/ TE = 7000/120 ms; gap = 1.3 mm; slices = 18; slice thickness = 6 mm; FA =110°; and voxel size = 0.65 × 0.95 × 6 mm^3^. SWI and FLAIR are used to observe traumatic lesions. SWI showed low intensity and FLAIR showed high intensity in traumatic lesions.

### MRI data preprocessing

Resting-state fMRI data preprocessing was performed using SPM12 software (http://www.fil.ion.ucl.ac.uk/spm/) implemented in MATLAB (version R2016b, MathWorks, Inc., Natick, MA, USA). The first ten scans were discarded to allow for magnetization equilibration, resulting in a total of 220 volumes. Resting-state data were realigned to the first volume to correct for inter-scan head motions; segmented into grey matter, cerebral spinal fluid, and white matter using the tissue probability maps; normalized into standard Montreal Neurological Institute template using nonlinear transformations and spatially smoothed with a Gaussian kernel of 6 mm full-width at half-maximum.

### Group ICA

**Fig. 1:**
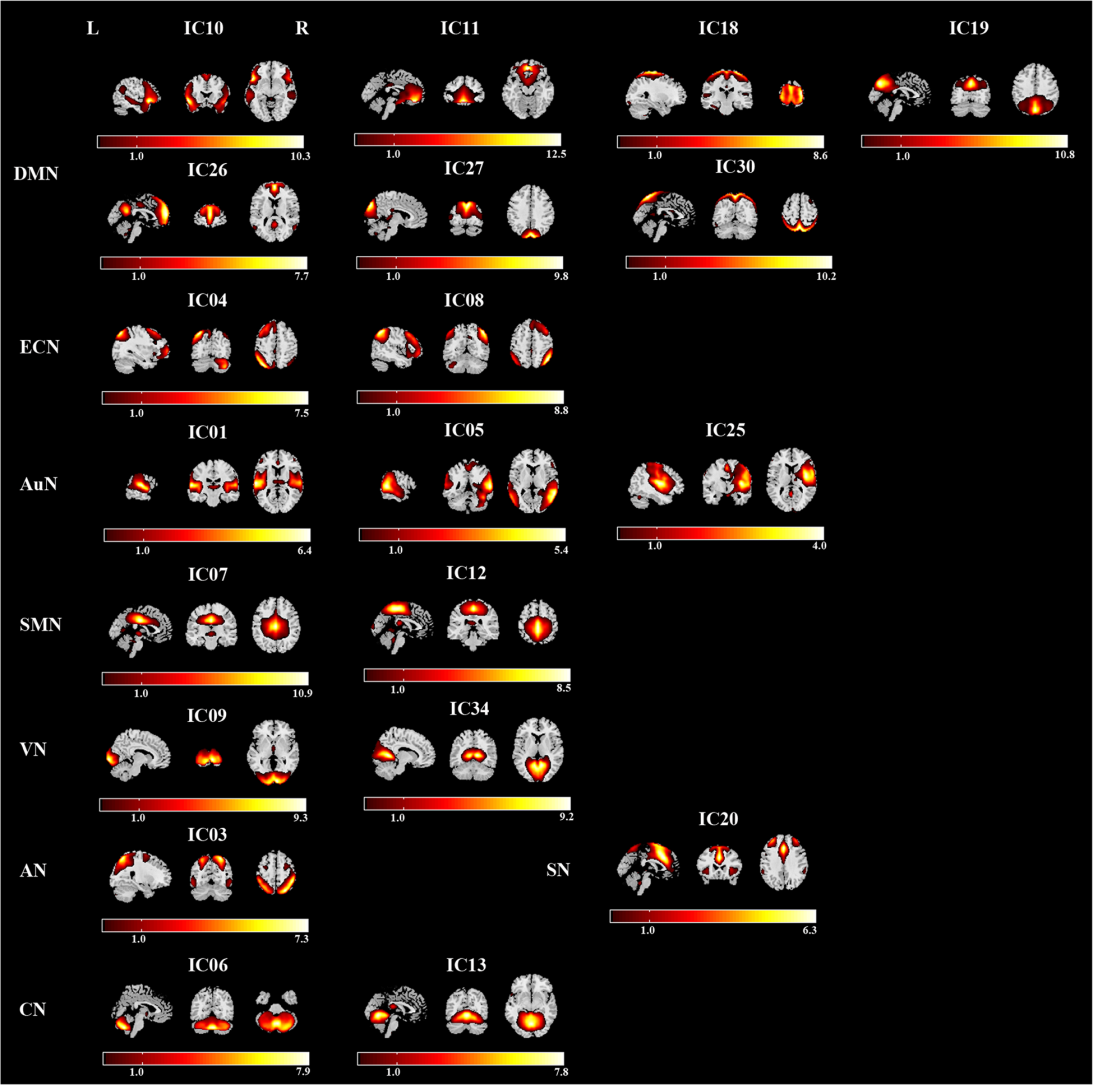
Spatial maps (displayed at the three most informative slices) of 20 independent components (ICs) that have chosen as our networks of interest. R represent right and L represents left. DMN, default mode network; SMN, sensorimotor network; ECN, executive control network; AuN, auditory network; AN, attention network; SN, salience network; VN, visual network; CN, cerebellum network

After data preprocessing, the data of all subjects were analyzed using the spatial independent component analysis method implemented by GIFT software[24, 25], and the data were decomposed into functional networks showing unique time history characteristics. The ICA analysis was performed in three stages: data reduction, application of the ICA algorithm, and back reconstruction for each individual subject. The number of independent components (ICs) was determined by using the minimum description length (MDL) criteria[26]. The data reduction was followed by a set of ICA, which was performed on the aggregate data of the subjects to produce an estimate of the ICA[24]. Then, the connectivity intensity value within each IC was converted into Z-score, reflecting the degree of correlation between the time series of a given voxel and the average time series of its corresponding components. To ensure the stability of the estimate, the algorithm was repeated 20 times in the ICASSO (http://research.ics.tkk.fi/ica/icasso/) algorithm, and the most central run was selected for further analysis. Spatiotemporal regression and regression reconstruction were used to obtain specific spatial maps and temporal processes of participants. Among the 34 components resulting from ICA, we selected 20 components (8 non-artifactual RSNs) as the focus of the subsequent analyses (Fig. 1) through visual inspection based on previous resting-state fMRI studies.

### SFNC analysis

The sFNC analysis was performed using the MANCOVAN toolbox in GIFT software to explore changes in the predefined 20 spatial IC pairs of functional connections. First, at 0.01-0.15 Hz, de-trend, de-peak and low-pass filtering were performed on the selected IC. Then, the pair correlations of these ICs were calculated and transformed using Fisher’s Z-transform. In the general linear model, static FNC group difference estimation was performed for each pair of resting state networks, controlling for age and gender. After multiple comparison adjustments for FDR, the significance threshold was *P* < 0.05.

### DFNC analysis

To understand the dynamic nature of FNC, we used the temporal dFNC toolkit in GIFT software. In order to calculate the dFNC between ICA time processes, a sliding window method was used, in which the convolution of a rectangle of width 20 and TRs = 40 s with a Gaussian (σ = 3 TRs) was progressively refined, with each step advancing 1 TR[27], resulting in W = 128 windows. The covariance between components was estimated according to the procedure outlined earlier[15]. Finally, the covariance matrices of each window were concatenated into a component × component × window array to represent the change of covariance (correlation) between networks (components) over time.

The dFNC windows with 500 iterations and 150 repeats were divided into five clusters by using the K-means clustering of square Euclidean distance realized by MATLAB [28]. The center of these clusters can be thought of as a small set of archetypes connecting “states“[29]. The number of optimal mental states was estimated using the elbow criterion (defined as the ratio of intra-cluster distance to inter-cluster distance). Using this method, k is 5 in the search window k is 2-10 [15]. To check the structure of dFNC states between groups, we evaluated the group level dFNC states.

### Differences between groups in dFNC and temporal properties

In each state differences between groups (mTBI+PTH and mTBI-PTH) in dFNC was investigated using two sample t-tests, and results were corrected for multiple comparisons using the false discovery rate (FDR) (*p*<0.05). We analyzed the temporal properties of dFNC states by computing the fractional windows and average dwell time in each state, as well as the number of transitions between states. Mean “dwell” time is defined as the number of contiguous windows belonging to a state, the “fractional windows” is the total number of windows belonging to a state, and the “number of transitions” is defined as the number of transitions between states, indicating the reliability of each state. Two-sample t test was used to analyze the significance of the mean residence time and transition times of each state in the mTBI +PTH group and the mTBI-PTH group (*p*<0.05, FDR corrected).

### Relationships between altered measurement and clinical variables in mTBI+PTH group

Pearson’s correlation analysis was used to analyze the correlation between changed network attributes (network metrics and temporal attributes) and clinical variables including MoCA score, headache frequency, and headache intensity, controlling for age and gender. SPSS 19.0 (IBM Corporation, Armonk, NY, USA) was used for statistical analysis, and *P* < 0.05 was used as the threshold.

## Results

### Demographic and clinical characteristics

The demographic and cognitive and headache characteristics of both mTBI patients with PTH group (mTBI+PTH) and mTBI patients without PTH group (mTBI-PTH) were shown in Table 1. There were no significant differences of age, gender, education, and MoCA performance between mTBI+PTH and mTBI-PTH group. Conventional imaging including SWI and FLAIR did not reveal any significant traumatic lesions.

**Table 1:** Demographic and cognitive variables of the mTBI with PTH patients and mTBI without PTH patients

Characteristics	mTBI+PTH (*n*=50)	mTBI-PTH (*n*=39)	*p*-value
**Age (years)**	38.42±11.38	42.36±10.75	0.088
**Education (years)**	13.10±3.02	12.49±3.08	0.350
**Gender (Female/ Male)**	22/28	15/24	0.668
**MoCA scores**	24.12±2.68	24.49±2.17	0.489
**Headache characteristics**			
**Predominant side**			
Right	19	-	-
Left	11	-	-
Bilateral	20	-	-
Unilateral	30	-	-
**Headache frequency**	11.74±7.18	-	-
**Headache intensity**	4.66±1.94	-	-

The data are shown as the mean ± SD. mTBI, mild traumatic brain injury; MoCA, Montreal Cognitive Assessment

### Networks of interests

From a total of 34 components, 20 Independent components (IC) were chosen as our networks of interest (Fig. 1), which were grouped into the following eight networks: default mode network (DMN) (ICs10,11,18,19,26,27,30), sensorimotor network (SMN)(ICs7,12), executive control network (ECN) (ICs4,8), auditory network (AuN) (ICs1,5,25), attention network (AN)(IC3), salience network (SN) (IC 20), visual network (VN) (ICs 9,34), cerebellum network (CN) (ICs 6,13).

### Differences between groups in sFNC

**Fig. 2:**
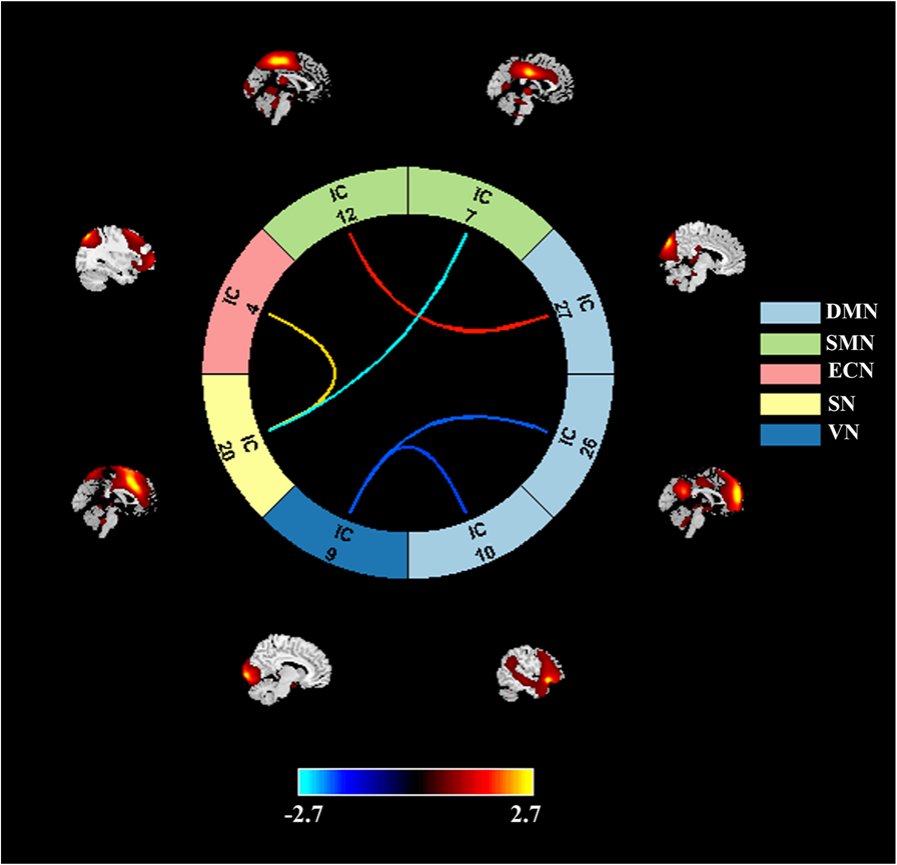
Significant differences in the network connectivity in the default mode network (DMN), sensorimotor network (SMN), executive control network (ECN), salience network (SN), visual network (VN) between mTBI+PTH group and mTB-PTH group

For the sFNC analysis, relative to the mTB-PTH group, the mTBI+PTH group exhibited significantly decreased interactions in two static connections, including the SN-SMN connection and VN-DMN connection. Moreover, compared with the mTB-PTH group, the mTB+PTH group also showed increased static FNC in the two interactions, including the SN-ECN and SMN-DMN. (Figure 2).

### Differences between groups in dFNC

**Fig. 3:**
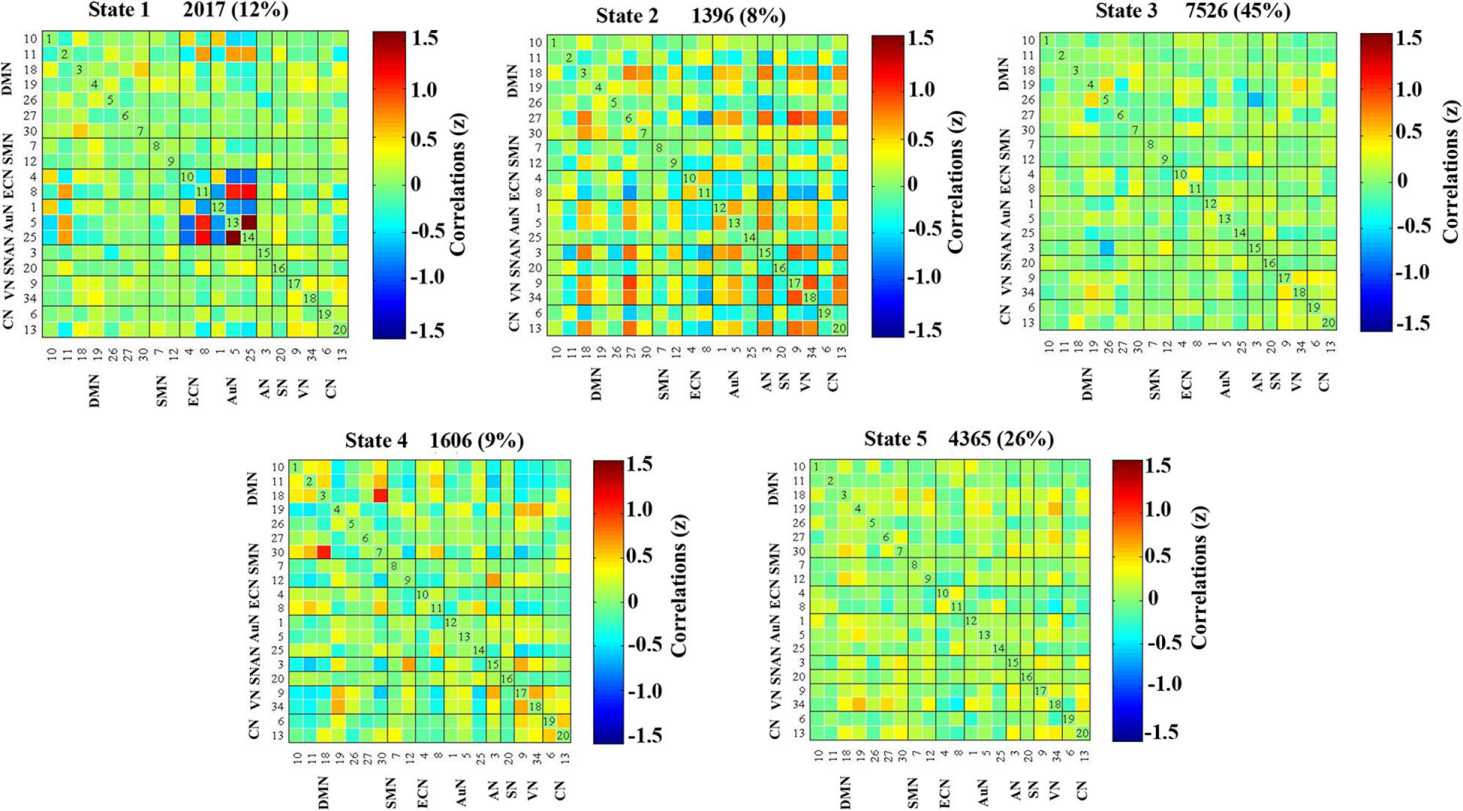
Results of the clustering analysis per state. Cluster centroids for each state. The total number of occurrences and percentage of total occurrences are listed above each cluster median

**Fig. 4:**
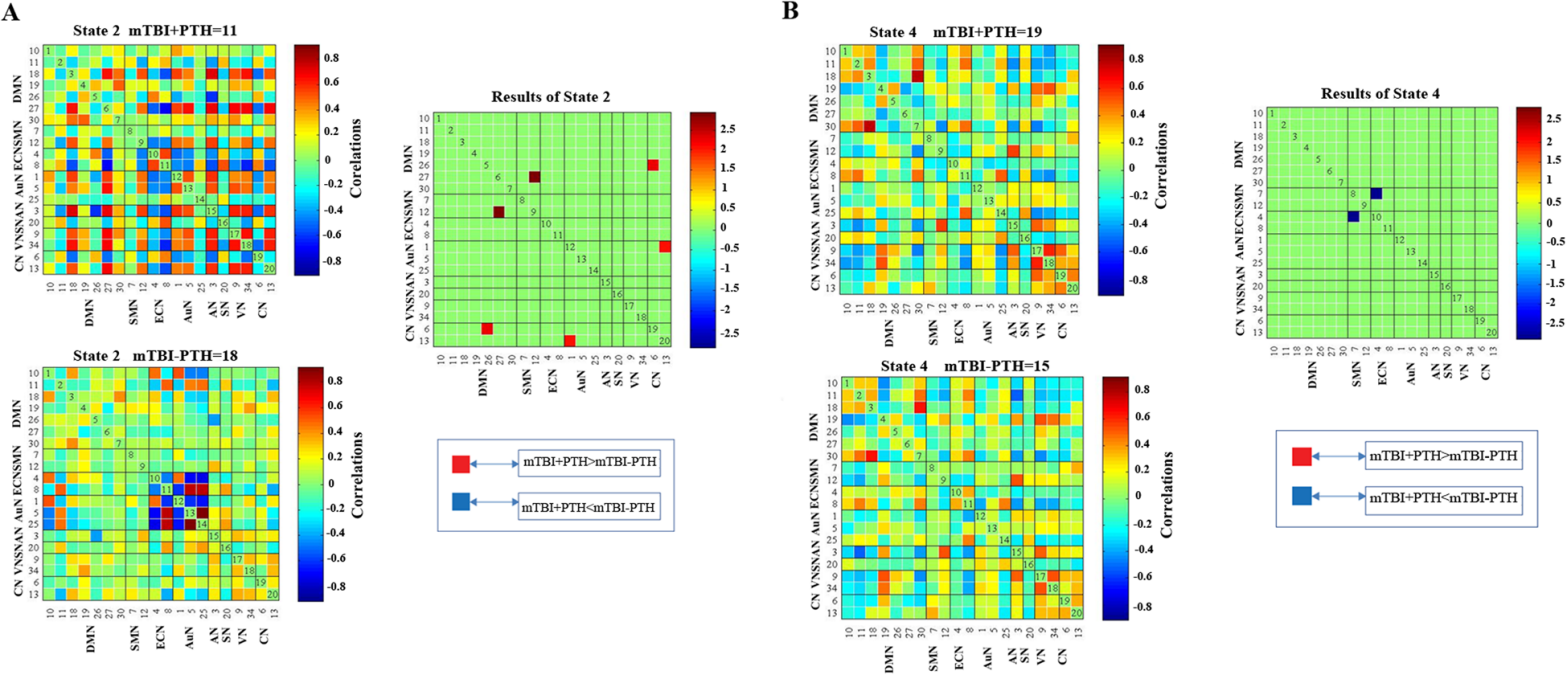
Differences in dynamic functional network connectivity(dFNC) between groups with (PTH) and without (PTH). DFNC assumes that whole brain connectivity sequentially iterates through a finite set of connectivity patterns known as dFNC states. Here only State 2 and state 4 are shown because significant differences between groups (PTH+ and PTH-) in dFNC were observed only in these states. The top and bottom row on the left side represents median connectivity matrices, called centroids (i.e. basis correlation patterns), in PTH+ and PTH- respectively. Figures also display the number of subjects that displayed correlation for at least ten windows and were included in the statistics to determine group differences

Using the k-means clustering method, we derived five highly structured states of FC that occurred repeatedly between individual scans and subjects. Figure 3 shows the five common functional connection states and corresponding cluster centers: the total percentages of these five states in all subjects were different, with State 1 (12 %), State 2 (8 %), State 3 (45 %), State 4 (9 %), State 5(26 %). Significant differences between groups in dFNC were observed only in state 2 and states 4 (Fig. 4). Connectivity among six networks differed between groups in state 2, two represented DMNs (IC26 and IC27); Two represented CN (IC6 and IC13); The other two networks represented AuN (IC1) and SMN (IC12) respectively. Three sets of coupling (DMN-SMN, DMN-CN, AuN-CN) differed between mTBI+PTH and mTBI-PTH group (mTBI+PTH > mTBI-PTH, *p*<0.05, FDR correction) (Fig. 4 A). In state 4, the between-network connections between DMN (IC26) and VN (IC9) differed between mTBI+PTH group and mTBI-PTH group (mTBI+PTH < mTBI-PTH, *p*<0.05, FDR correction) (Fig. 4B).

### Temporal properties of FC states

**Fig. 5:**
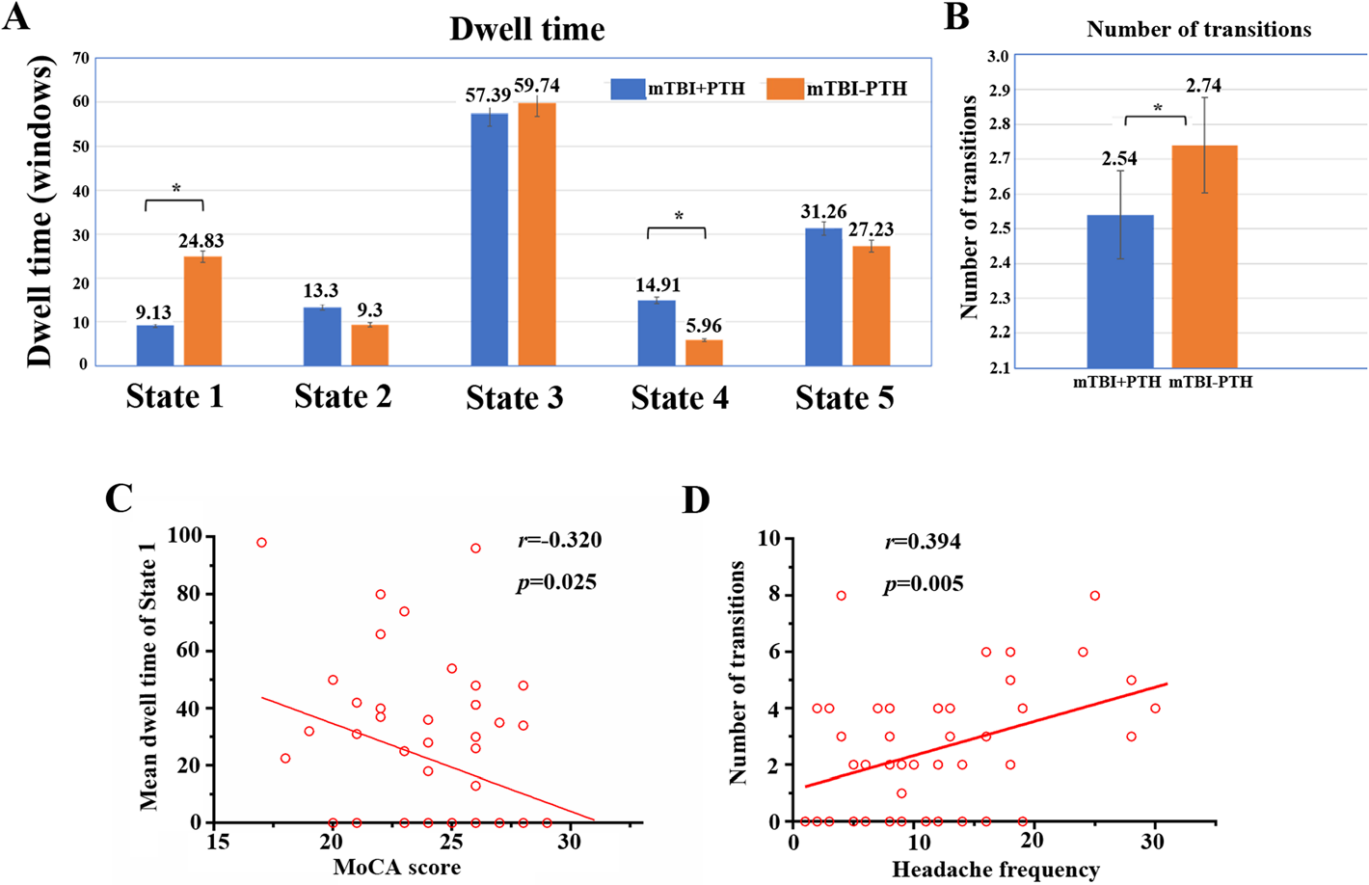
Temporal properties of functional connectivity state analysis and correlation of clinical variables with temporal properties for mTBI with PTH patients. **A **mean dwell time (i.e. number of consecutive windows spent in each state before switching) and (**B)** number of transitions (i.e. switching between states) is depicted for mTBI with PTH and mTBI without PTH with error bars. Asterisk represent a significant group difference (*p*<0.05, FDR corrected). **C **the mean dwell time of state 1 was negatively correlated with MoCA score. **D **the number of state transitions was positively associated with the headache frequency in mTBI with PTH patients (all *p*<0.05, FDR corrected)

As shown in Fig. 5, significant group differences were identified in the mean dwell time of two states. Specifically, the mean dwell time in state 1 was significantly shorter in mTBI+PTH group compared to mTBI-PTH group (mTBI+PTH: 9.13±15.58; mTBI-PTH: 24.83±34.09, *p*<0.05). In contrast, the mean dwell time in state 4 was significantly longer in mTBI +PTH group compared with mTBI-PTH group (mTBI +PTH: 14.91±21.93; mTBI-PTH:5.96±11.30, *p*<0.05). In addition, the number of transitions between states in mTBI +PTH group was smaller than in mTBI-PTH group (mTBI+PTH: 2.54±2.22; mTBI-PTH: 3.64±2.46, *p*<0.05). However, we did not find any significant group differences in fractional windows in each state (all *p*>0.05).

### Correlation results

The correlation between FC attributes and cognitive performance and headache measurements in the mTBI +PTH group was further analyzed (Fig. 5). We found that the number of transitions between states was positively associated with headache frequency (*r*=0.394, *p*=0.005), indicating the relationship between dynamic states changes and mTBI patients’ headache frequency. Additionally, dwell time in State 1 was negatively correlated with MoCA score (*r* = -0.320, *p*=0.025).

## Discussion

The present study combined sFNC and dFNC analyses to investigate the whole brain features of mTBI with PTH with a focus on the FNC states as well as the temporal properties. Our results revealed the sFNC and dFNC features and altered dynamic temporal properties, moreover, the temporal characteristics is associated with the headache measurements and cognitive performance in mTBI+PTH group.

For sFNC, our results showed that abnormal interactions of DMN-VN, DMN-SMN in mTBI with PTH patients, which are partly in line with prior neuroimaging results in mTBI with PTH and migraine patients[8, 30]. The particular focus of mTBI patients on self-related symptoms, known as PTH, may uniquely affect brain networks involved in pain perception and regulation. Notably, attention to pain or pain diversion has emerged as an important cognitive regulatory mechanism to explain pain perception in acute and chronic diseases[31]. We hypothesize that the association between cognitive control of the higher order cortex and hypersensitivity to pain sensation may be disrupted and may contribute to PTH after mTBI. Despite the similarity, mTBI with PTH patients also showed specific abnormalities in sFNC, such as decreased FNC in SN-SMN, and increased FNC in SN-ECN pairs. Our result provided further evidence that the insula, a main area belonging to the SN, plays a specific role in pain regulation and emotional experience from information about body states[32]. Moreover, the pain can activate both the insula and the medial cingulate cortex whether acute or chronic, psychological or physical [33].

In comparing dFNC differences within RSNs between groups, we found significant between-group FNC differences among several RSNs, including the DMN, SMN, CN, AuN and VN. Of note, the disrupted FNC in state 2 and state 4 was primarily related to the DMN, which is considered to be an endogenous neural network specialized for self-referential thinking and introspection [34]. Several studies on chronic pain have shown both increased and decreased cortical activity in brain regions within the DMN, and dysregulation of this network is thought to represent neuroplasticity in the brain during nerve repair and recovery after injury [35–37]. In line with these reports, we found aberrant dFNC between the DMN and other networks in mTBI+PTH group. Disrupted dFNC between the DMN and other networks could serve as a potential biomarker for neurocognitive dysfunction and PTH after mTBI. Additionally, recent researches have shown that the cerebellum is also involved in a variety of functions, including cognitive and affective processing, pain-related processing, athletic-related processing, and thirsty experience[38, 39]. Similarly, our results showed disrupted dFNC between CN and DMN as well as AuN, suggesting that the PTH after mTBI may be related with the disruption of CN. Therefore, we hypothesize that the association between hypersensitivity to pain and higher-order cortical cognitive control may be disrupted and as a whole involved in PTH following mTBI, and the results of FNC improve our understanding of the pathophysiological mechanisms of PTH in mTBI patients.

MTBI patients with PTH have significantly different dFNC temporal properties overall. The former dwelled different in the state 1 and state 4, and showed a lower number of transitions to the strongly interconnected states. Thus, the reduced transition between brain states may be used for differentiating mTBI with PTH from mTBI without PTH. In addition, the temporal properties of dFNC are linked to cognitive outcome in multiple domains, such as memory, attention, execution and visuospatial function [16]. Zou et al. conducted a correlation analysis between dynamic temporal properties and clinical characteristics of headache, and observed a positive correlation between the changes in transition times between different states and the headache severity of chronic migraine[37]. Anyway, these observations may indicate the vulnerability of resting-state functional networks in mTBI with PTH, as well as emphasize the importance of exploring PTH after mTBI for the temporal dynamic FC. Therefore, the temporal properties of dynamic FC might help elucidate the neuropathological basis of the PTH, and serve as a potential imaging biomarker for investigating and predicting the PTH. Furthermore, the current findings might help clinicians to make therapeutic strategy for PTH.

There are some limitations in the current study. First, this is a preliminary cross-sectional study of dFNC changes in PTH after mTBI, and the sample size is limited, making it difficult to directly infer a causal relationship between the brain functional network of PTH status and cognitive impairment after mTBI. Longitudinal studies with larger samples are required. Second, this study only includes mTBI patients with and without PTH, but not taking health controls into consideration. Future studies including these three groups will be needed. Third, it has been suggested that dFNC analysis should be performed after at least 10 min of resting state acquisition[40]. Our resting state acquisition time was 8 min, which allowed stable resting-state fMRI data to be obtained. Moreover, the time between injury and MRI scanning is not consistent, which may have effect on the results. Finally, there was no scale measuring neuropsychological states, including depression and anxiety during headaches, therefore, the influence of these neuropsychological factors on FNC has not been assessed.

## Conclusions

This study explored for the static and dynamic FNC patterns in mTBI patients with PTH. Compared to mTBI patients without PTH, the temporal properties of functional dynamics (number of transitions and dwelling time) were altered in mTBI patients with PTH, which were correlated with cognitive performance and headache characteristics. Taken together, the FNC and the temporal properties of dynamic FC might help elucidate the neuropathological basis of the PTH, and serve as a potential imaging biomarker for investigating and predicting the PTH to determine more appropriate management.

## Data Availability

Clinical, neuroimaging and statistical data will be available upon request from any qualified investigator.

## Abbreviations

fMRI: functional magnetic resonance imaging
PTH: post-traumatic headache
mTBI: mild traumatic brain injury
FC: functional connectivity
sFNC: static functional network connectivity
dFNC: dynamic functional network connectivity
DMN: default mode network
CN: cerebellum network
SMN: sensorimotor network
AuN: auditory network
VN: visual network
AN: attention network
ECN: executive control network
GCS: Glasgow Coma Score
MoCA: Montreal Cognitive Assessment
VAS: visual analogue scale
MDL: minimum description length
